# Odontological Society of Pennsylvania

**Published:** 1881-05

**Authors:** 


### ARTICLE III.

### *The Odontological Society of Pennsylvania.*

The regular meeting of the Odontological Society of Pennsylvania was held Saturday evening, February 5th, 1881, at the office of Dr. Pettit, 1624 Chestnut Street, Dr. Dan'l Neall in the chair.

Dr. E. R. Pettit exhibited and explained his improvement of the dental engine, which is a very simple device, especially applicable to the S. S. White dental engine, and it can be attached to it in a few minutes' time. This improvement consists of a hinged joint with a swivel movement, which is inserted between the standard of the dental engine and its attachment to the base, above the driving wheel. By means of this joint—which is simply a friction clutch—the standard may be changed *instantly*, with one hand, to any desired angle, the swivel movement permitting the top of the standard to follow the slightest movement of the handpiece back and forth. As with this movement the rocking motion of the standard is not desirable, he also exhibited a simple arrangement which accompanies the joint, by which the rocking was prevented. He claims that by this simple device the *drag* upon the hand is *entirely avoided*, thns permitting greater delicacy of touch; much greater freedom of movement of the handpiece itself; avoiding the vibration of the flexible arm, and consequent breakage of disks; and also by means of an extension treadle, or the use of a motor, the dentist can operate equally well on either side of the chair without moving the engine, as the top of the standard is brought directly in front of the patient.

He also exhibited his new device for holding the engine arm in place when not in use. It consists of a ring of metal clamped to the sleeve above the handpiece and a hook soldered to the standard, about fourteen inches from its top. The ring is to be placed upon the hook when the engine is not in use.

Dr. Pettit also exhibited Griscom's new electro-motor for sewing machines and dental engines. He explained its construction, and demonstrated its many advantages over previous ones in power, simplicity and size, as it weighs but three and one-half pounds. He showed his method of attaching it to the dental engine, and a simple means by which he could change from the motor to the treadle, or

*vice versa*, in half a minute, when his hinged joint is used. The battery is contained in a box about 18x18x12 inches in size, and consists of six cells. There is a simple device by which the zincs are raised from the acid when the motor is not in use, so that there is no loss from local action. By this same arrangement the speed of the sewing machine or dental engine is regulated, and is under perfect control. It is supposed that for dental purposes one charge of this battery will last about six months. As there are no fumes from it, it is kept in the office without danger of injury to instruments, &c.

Dr. J. A. Woodward.—The motor, as arranged by Dr. Pettit, will do all that is required, safely and as conveniently as any motor available at present in Philadelphia. For my use a properly constructed foot-engine is preferred. Eclectrical appliances exact uniform conditions to work efficiently. Their care cannot be given to any but those who understand and have sufficient interest to keep them in proper order. The advantages of the motor would not be sufficient to compensate me for the care it would demand.

Dr. Bonwill thought that the best motor of the day, all things considered, and the proper one for us, was the water motor in some form. He thought our attention should be directed to the perfecting of this class of motors. Electro-motors, he thought, were too intricate and expensive, and liable to get out of order, while the water motor was without valve; simple and easily managed by even the least intelligent.

Dr. E. R. Pettit spoke in favor of electro-motors over steam, water or other motors; that their running cost is but little; they are always ready for use; easily moved from place to place; comparatively noiseless; under perfect control; require less care than any other kind of motor, and when properly made are much less likely to get out of order. It must not be supposed that they are delicate instruments, like the electric mallet, for, he claimed, that there is literally nothing to get out of order, with proper care. They will last until worn out.

Dr. Bonwill looked forward to good results from the "Otto" silent gas engine, and also to a French gas engine, recently illustrated in the *Popular Science Monthly*. Had been interested recently in the "Tyson" motor, but did not believe it suitable for a dental office on account of the smell of steam, hot oil, &c. He called on Dr. Webb to corroborate what he had said in regard to the difficulty of keeping the electric battery in order, and of the few persons who had proven themselves capable of successfully managing one.

Dr. Webb stated, that while few understand and properly operate the electro-magnetic mallet and charge the battery, still, thorough knowledge of a Bunsen cell is easily acquired, and when each is properly charged, and the four cells are carefully and tightly connected, the battery is always in order till it weakens, and must be recharged. It requires only about fifteen minutes to wash out and charge two of the four cells, alternately, each week, and the battery costs, on the average, about twenty-five cents per week. Because so many practitioners do not attend to or put the battery in order, this does not argue against the instrument, since nearly all the finest operators are successfully and daily using it.

Dr. Kingsbury spoke of his early experiments with electricity in connection with odontalgia and the extraction of teeth by means of the fluid. He regretted the evident tendency to the introduction of machinery into the dental office. He thought the increasing tendency in the direction of more machinery was to be depreciated. He wished to enter his protest against any further extension in this direction, as he believed that too much machinery only tended to alarm our patients, and render the dental office more repulsive to them. The dental engine was a valuable acquisition, he would acknowledge, and in the hands of the careful operator was a benefit, but in the hands of an ignorant or careless person it was capable of doing great harm, as it had done already. He believed he had no use for electricity or steam as a motive power, either for excavating or filling teeth.

Dr. Merriman, of Salem, Mass., being present and called upon, spoke in favor of the electric mallet, and stated that the patient's dread of the dentist to-day was not nearly as great as formerly.

Dr. Kingsbury said that he frequently saw fillings done twenty years ago that were as good as any done by the electric mallet to-day.

Dr. Webb felt sure that no fillings had ever been inserted according to the old, or any other methods, where the gold was as uniform in density, or made as compact and as susceptible to a fine finish, and where it was so placed against and over finely prepared edges and frail walls of enamel, so easily, rapidly and perfectly as is done to-day by the best operators with the electro-magnetic mallet. There is no such thing as mere mechanical manipulation; each definite movement of the hand which guides an instrument is preceded by a definite movement of the molecules of living matter in ganglionic elements of the brain.

Dr. James Truman was opposed to the sentiment that would stop all efforts toward progress. He regarded the so-called machines of the day as a great advance over the past; but at the same time he could not appreciate the value of motors in "engine" work. The labor of running this machine was comparatively light, and we needed the intelligence that could only be secured by the operator attending to it himself. The rapid changes in motion could not well be secured by any motor, however perfect. He was satisfied by experience and experiment that no instrument consolidated gold equally as well as the electric mallet, but owing to difficulties to him insurmountable, its use had been abandoned. It is possible that in its present more perfect condition these objections may have lost their force.

Dr. Bonwill could not sit still and listen to the anathemas of the president and Dr. Kingsbury against the mechanical appliances of the day, without offering his testimony in favor of many of the devices that are now used that ten years ago were scarcely thought of. While he recognized them as great blessings, yet many operators failed to apply them to the full benefit of the patient in the saving to him of money and time. The electric mallet could be made a very great nuisance; yet, when intelligently manipulated, it could be made to work wonders, and be a blessing to patient and operator, although he preferred his mechanical mallet, believing it far superior, and no labor to keep in order or to kick it. Were he obliged to desist from the use of these appliances he would give up the practice of dentistry. Instead of driving patients away he believed his practice was not in the least curtailed from the persistence in their use. He does not use the mallet as much as formerly, believing gold alloy superior to gold alone, for many of the cases pertaining to the cuspids.—*Dental Office and Laboratory.*

